# *N*,*N*′-Bis[tris­(hy­droxy­meth­yl)meth­yl]propane-1,3-di­amine (bis-tris propane)

**DOI:** 10.1107/S241431462500954X

**Published:** 2025-11-06

**Authors:** Ismael Angel-Nieto, Rosa Elena Arroyo-Carmona, Aarón Pérez-Benítez, Sylvain Bernès

**Affiliations:** a Instituto de Física Luis Rivera Terrazas, Benemérita Universidad Autónoma de Puebla, Av. San Claudio y 18 Sur, 72570 Puebla, Pue., Mexico; b Facultad de Ciencias Químicas, Benemérita Universidad Autónoma de Puebla, Ciudad Universitaria, 72570 Puebla, Pue., Mexico; Goethe-Universität Frankfurt, Germany

**Keywords:** crystal structure, supra­molecular structure, hydrogen bonds, polymorphism propensity

## Abstract

The title compound, C_11_H_26_N_2_O_6_, used for the preparation of buffer solutions and high-nuclearity coordination complexes, crystallizes with a half mol­ecule in the asymmetric unit. The full mol­ecule is completed through mirror symmetry *m* in the space group *Pnma*. The mol­ecular shape is bent and, as a consequence, some H atoms are disordered to avoid too short H⋯H intra­molecular contacts. Mol­ecules in the crystal are linked *via* O—H⋯N and N—H⋯O hydrogen bonds, forming chains along [100], which are further packed through other O—H⋯O hydrogen bonds between hy­droxy groups. The here-reported structure probably represents the less-stable form in a set of polymorphs.

## Abstract 2

The crystal structure of a reagent frequently used for the preparation of buffer solutions is reported, showing that the mol­ecule lies on a crystallographic mirror plane, and has a bent conformation.

## Graphical abstract

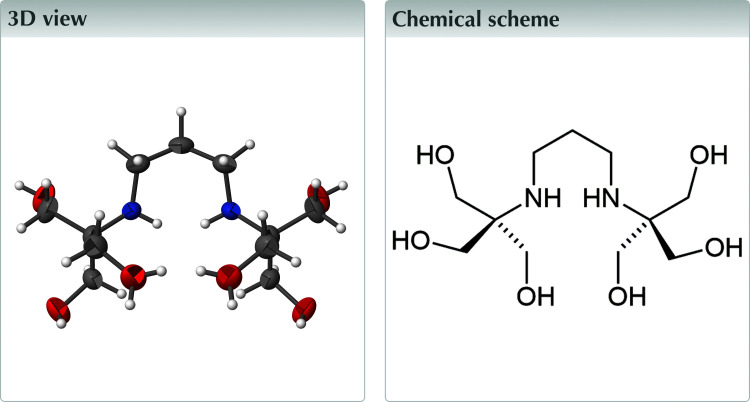

## Structure description

**Figure 1:**
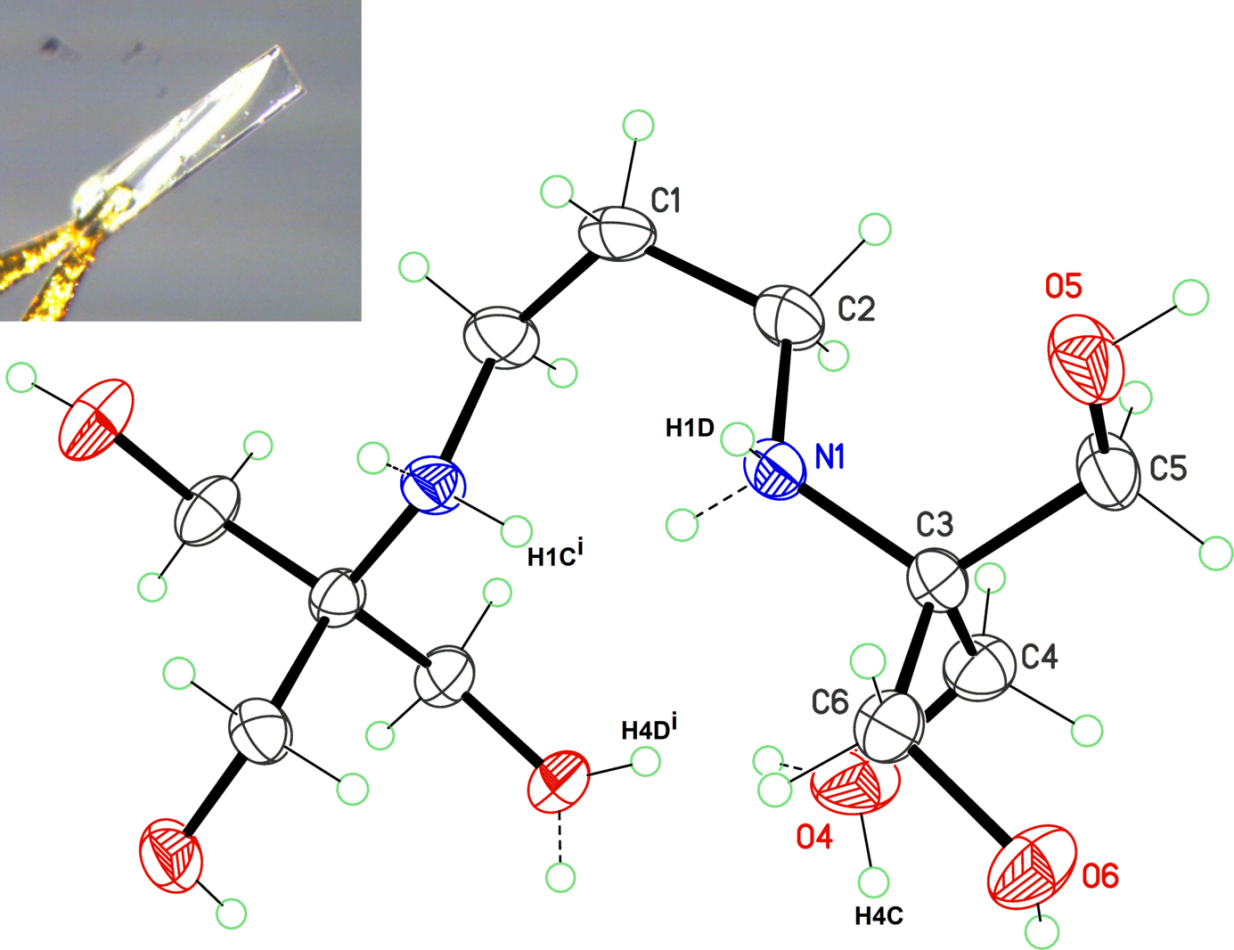
Mol­ecular structure of the title compound, with displacement ellipsoids for non-H atoms at the 30% probability level. H atoms with dashed bonds are disordered counterparts for crystallographically equivalent H atoms generated through *m* symmetry (*x*, 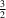 − *y*, *z*). Non-labelled C, N and O atoms are generated using the same mirror symmetry. The top-left inset shows the single crystal used for data collection. It is *ca*. 0.7 mm long.

1,3-Bis[tris­(hy­droxy­meth­yl)methyl­amino]­propane, also known as bis-tris propane or BTP, is a di­amino­polyol used in biochemistry and mol­ecular biology for the preparation of buffer solutions in the wide pH range 6.0–9.5. It is readily soluble in water, and can be recrystallized as large plate-shaped single crystals (Fig. 1 ▸, inset).

This polydentate mol­ecule can also be used as a ligand for coordination chemistry. Crystal structures based on Cu^2+^ (*e.g*. Milway *et al.*, 2013 ▸; Kirillova *et al.*, 2017 ▸), V^4+^ (Nachtigall *et al.*, 2017 ▸), polyoxidomolybdates (Li *et al.*, 2015 ▸) and lanthanides (*e.g*. Yinling *et al.*, 2022 ▸) have been reported. It is surprising that the crystal structure of the free ligand BTP has never been published.

BTP crystallizes in a centrosymmetric space group, *Pnma*, with the mol­ecule placed on the mirror plane normal to the unit-cell *b* axis (Fig. 1 ▸). Within this structure, the mol­ecule thus belongs to the *C_s_* point group, although it does not display a *trans*-extended geometry, as might be expected. Instead, it adopts a bent-shaped geometry, defined by the *gauche* torsion angle N1—C2—C1—C2^i^ = −70.4 (3)° [symmetry code: (i) *x*, 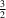 − *y*, *z*]. This shape was previously observed for a Ca^2+^ complex (Liu *et al.*, 2021 ▸) or in Cu^2+^ coordination compounds (Milway *et al.*, 2013 ▸). The amine H atom is clearly disordered over two equally occupied positions, H1*C* and H1*D*, avoiding a short H⋯H contact [H1*C*⋯H1*C*^i^ ≃ 1.4 Å], which would be destabilizing for the mol­ecular structure. In the same way, the H atom for the hy­droxy group O4 is disordered over two sites, H4*C* and H4*D*. An alternative would be to refine a non-disordered model in space group *Pn*2_1_*a*, with independent amine H atoms fully occupying sites H1*C* and H1*D* on two independent N atoms, as well as hy­droxy H atoms with full occupancy on two independent O4 atoms. Such a model refines well (*R*_1_ = 0.039) but is unlikely, for two reasons: (i) convergence is not reached if H atoms are refined with free coordinates, and (ii) intensity statistics show a centric distribution, with, for example, <|*Z* − 1|> = 0.957 (theoretical: 0.968). These disordered H atoms also allow the formation of two intra­molecular N—H⋯N and O—H⋯O hydrogen bonds, consolidating the bent conformation (Table 1 ▸, entries 1 and 2).

**Figure 2:**
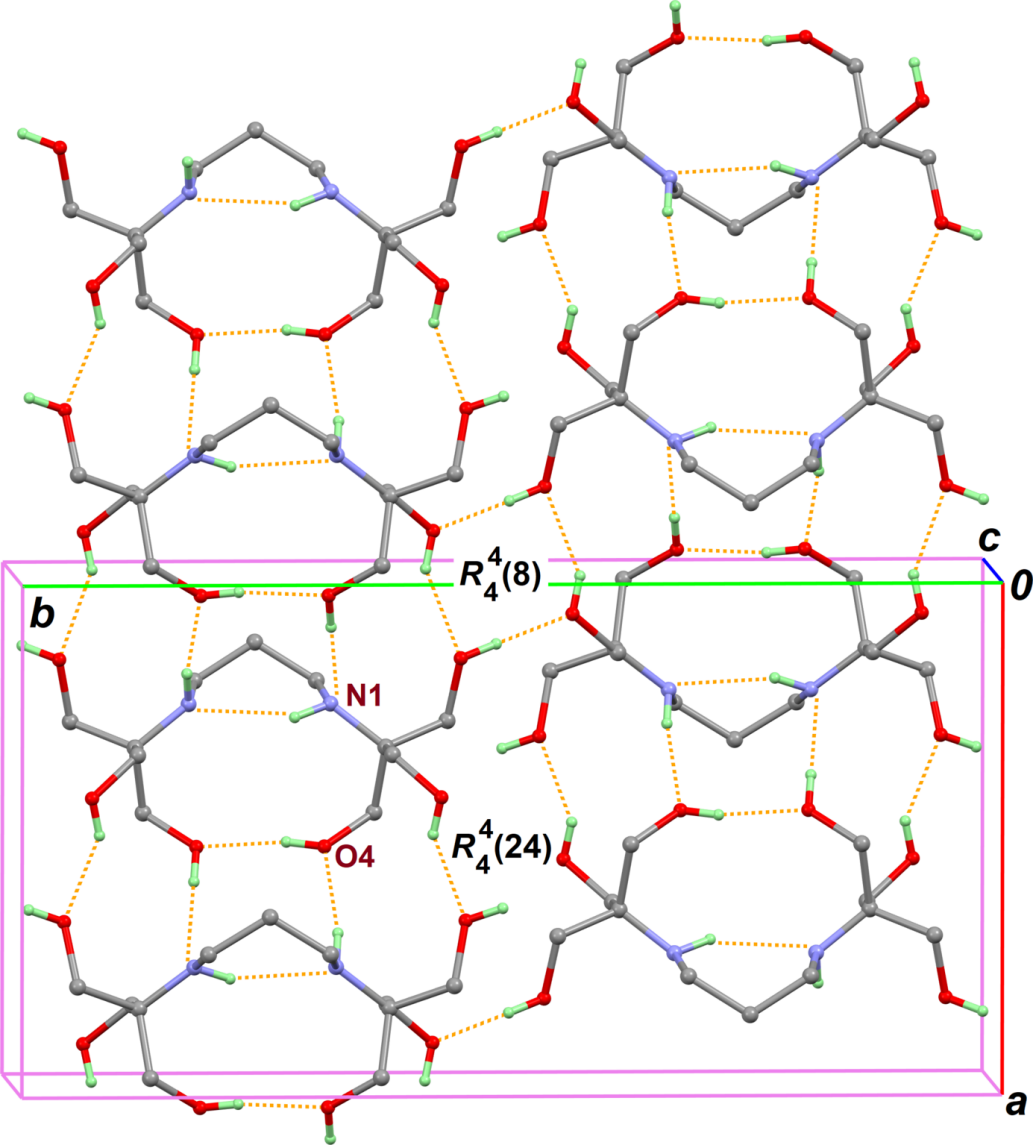
Part of the crystal structure, as viewed down unit-cell axis *c*, showing the framework of hydrogen bonds (orange dashed lines). For the sake of clarity, only one position for disordered H atoms bonded to amine N1 and hy­droxy O4 groups is retained, and all C-bonded H atoms are omitted.

The crystal structure is essentially monoperiodic: the mol­ecules form chains along the [100] direction through the inter­molecular hydrogen bonds O4—H4*C*⋯N1^ii^ and N1—H1*D*⋯O4^iii^ involving amine and hy­droxy functional groups with disordered H atoms (Table 1 ▸, entries 3 and 4). Inter­chain O—H⋯O contacts build a network of fused centrosymmetric 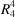
(8) and 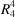
(24) ring motifs, parallel to the monoperiodic chains (see two last entries in Table 1 ▸ and Fig. 2 ▸).

**Figure 3:**
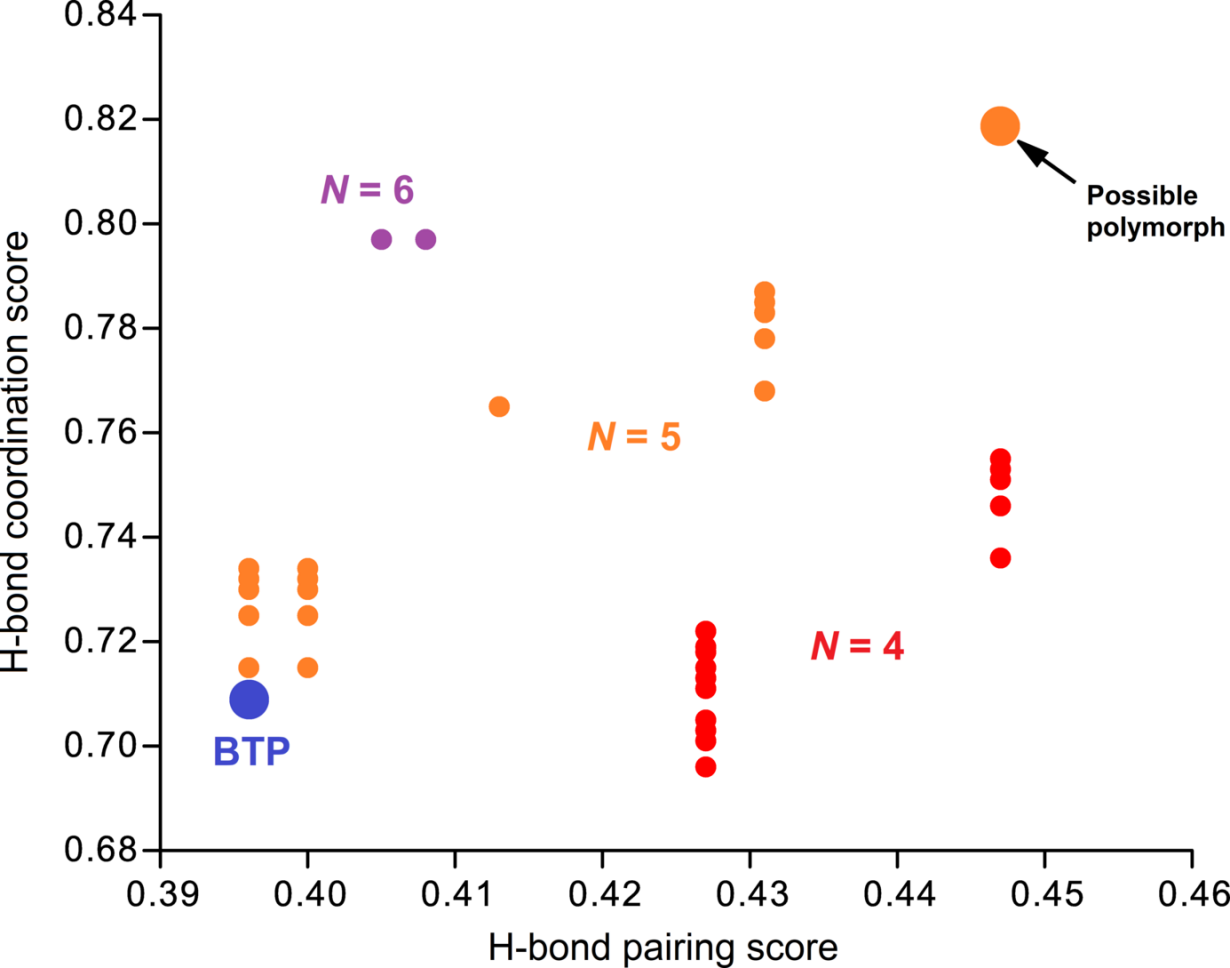
Hydrogen-bonding landscape for BTP, calculated with *Mercury* (Macrae *et al.*, 2020 ▸). The blue dot on the left-bottom corner corresponds to the here-reported structure, while the orange spot on the upper-right corner is for an hypothetical polymorph. *N* represents the number of inter­molecular hydrogen-bond pairs, which are shown with different colours. For the regression analysis, the area under ROC curve was 0.835. Putative hydrogen-bonding networks were built with likelihood > 0.1, for both hydrogen-bond propensity and hydrogen-bond coordination.

The unexpected conformation reported herein for BTP could be a consequence of a propensity to polymorphism. The assessment of hydrogen-bond coordination likelihood of BTP was carried out using the hydrogen bond propensity tool available in *Mercury* (Galek *et al.*, 2014 ▸; Macrae *et al.*, 2020 ▸), with the CSD-6.00 database as a training dataset. The model refined in the *Pn*2_1_*a* space group was used as a target, since the mol­ecule is disorder-free, and the asymmetric unit includes the complete BTP mol­ecule (*Z*′ = 1). The resulting hydrogen-bonding landscape is compelling (Fig. 3 ▸): in the map (mean hydrogen-bond pairing propensity, mean hydrogen-bond coordination), the here-reported crystal structure is found at coordinates (0.396, 0.709), while a more stable polymorph is predicted at coordinates (0.447, 0.819). We thus assume that the Ostwald’s rule for the formation of polymorphs holds, and that we crystallized the less-stable form of BTP.

## Synthesis and crystallization

BTP, coming from a commercial supplier (Sigma-Aldrich), was recrystallized from a saturated water solution, at room temperature. Single crystals were obtained after a few days.

## Refinement

Crystal data, data collection and structure refinement details are summarized in Table 2 ▸. All H atoms were visible in difference maps, and were refined with free coordinates and isotropic displacement parameters. O—H and N—H bond lengths were restrained to 0.85 (2) and 0.90 (2) Å, respectively. H atoms bonded to N1 and O4 are disordered over two positions (H1*C*/H1*D* and H4*C*/H4*D*), and their occupancies were fixed to 1/2.

## Supplementary Material

Crystal structure: contains datablock(s) I, global. DOI: 10.1107/S241431462500954X/bt4185sup1.cif

Structure factors: contains datablock(s) I. DOI: 10.1107/S241431462500954X/bt4185Isup2.hkl

Supporting information file. DOI: 10.1107/S241431462500954X/bt4185Isup3.cml

CCDC reference: 2498903

Additional supporting information:  crystallographic information; 3D view; checkCIF report

## Figures and Tables

**Table 1:** Hydrogen-bond geometry (Å, °)

*D*—H⋯*A*	*D*—H	H⋯*A*	*D*⋯*A*	*D*—H⋯*A*
N1—H1*C*⋯N1^i^	0.86 (2)	2.24 (3)	2.970 (3)	143 (3)
O4—H4*D*⋯O4^i^	0.80 (3)	1.90 (3)	2.679 (3)	162 (6)
O4—H4*C*⋯N1^ii^	0.82 (3)	2.09 (3)	2.905 (2)	175 (4)
N1—H1*D*⋯O4^iii^	0.82 (2)	2.09 (3)	2.905 (2)	169 (3)
O6—H6⋯O5^ii^	0.81 (2)	2.02 (3)	2.7390 (19)	148 (3)
O5—H5⋯O6^iv^	0.85 (2)	1.88 (2)	2.7305 (18)	176 (3)

Symmetry codes: (i) 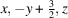; (ii) 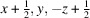; (iii) 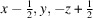; (iv) 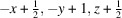.

**Table 2:** Experimental details

Crystal data	
Chemical formula	C_11_H_26_N_2_O_6_
*M*_r_	282.34
Crystal system, space group	Orthorhombic, *Pnma*
Temperature (K)	296
*a*, *b*, *c* (Å)	10.7262 (3), 20.5189 (5), 6.4624 (3)
*V* (Å^3^)	1422.31 (8)
*Z*	4
Radiation type	Ag *K*α, λ = 0.56083 Å
μ (mm^−1^)	0.07
Crystal size (mm)	0.68 × 0.15 × 0.05

Data collection	
Diffractometer	Stoe Stadivari
Absorption correction	Multi-scan (*LANA*; Stoe, 2025 ▸)
*T*_min_, *T*_max_	0.960, 0.997
No. of measured, independent and observed [*I* > 2σ(*I*)] reflections	68162, 1942, 1392
*R*_int_	0.058
(sin θ/λ)_max_ (Å^−1^)	0.682

Refinement	
*R*[*F*^2^ > 2σ(*F*^2^)], *wR*(*F*^2^), *S*	0.047, 0.140, 1.08
No. of reflections	1942
No. of parameters	150
No. of restraints	6
H-atom treatment	All H-atom parameters refined
Δρ_max_, Δρ_min_ (e Å^−3^)	0.25, −0.22

Computer programs: *X-AREA Pilatus3-SV*, *X-AREA Recipe*, *X-AREA Integrate* and *LANA* (Stoe, 2025 ▸), *SHELXT2018/2* (Sheldrick, 2015*a* ▸), *SHELXL2025/1* (Sheldrick, 2015*b* ▸), *XP* in *SHELXTL-Plus* (Sheldrick, 2008 ▸), *Mercury* (Macrae *et al.*, 2020 ▸) and *publCIF* (Westrip, 2010 ▸).

## full crystallographic data

### N,N'-Bis[tris(hydroxymethyl)methyl]propane-1,3-diamine Crystal data

**Table:**

C_11_H_26_N_2_O_6_	*D*_x_ = 1.319 Mg m^−^^3^
*M**_r_* = 282.34	Melting point: 437 K
Orthorhombic, *P**n**m**a*	Ag *K*α radiation, λ = 0.56083 Å
*a* = 10.7262 (3) Å	Cell parameters from 34115 reflections
*b* = 20.5189 (5) Å	θ = 2.9–26.7°
*c* = 6.4624 (3) Å	µ = 0.07 mm^−^^1^
*V* = 1422.31 (8) Å^3^	*T* = 296 K
*Z* = 4	Plate, colourless
*F*(000) = 616	0.68 × 0.15 × 0.05 mm

### N,N'-Bis[tris(hydroxymethyl)methyl]propane-1,3-diamine Data collection

**Table:**

Stoe Stadivari diffractometer	1942 independent reflections
Radiation source: Sealed X-ray tube, Axo Astix-f Microfocus source	1392 reflections with *I* > 2σ(*I*)
Graded multilayer mirror monochromator	*R*_int_ = 0.058
Detector resolution: 5.81 pixels mm^-1^	θ_max_ = 22.5°, θ_min_ = 3.0°
ω scans	*h* = −14→12
Absorption correction: multi-scan (*LANA*; Stoe, 2025)	*k* = −28→28
*T*_min_ = 0.960, *T*_max_ = 0.997	*l* = −8→8
68162 measured reflections	

### N,N'-Bis[tris(hydroxymethyl)methyl]propane-1,3-diamine Refinement

**Table:**

Refinement on *F*^2^	Primary atom site location: dual
Least-squares matrix: full	Secondary atom site location: difference Fourier map
*R*[*F*^2^ > 2σ(*F*^2^)] = 0.047	Hydrogen site location: difference Fourier map
*wR*(*F*^2^) = 0.140	All H-atom parameters refined
*S* = 1.08	*w* = 1/[σ^2^(*F*_o_^2^) + (0.0665*P*)^2^ + 0.2802*P*] where *P* = (*F*_o_^2^ + 2*F*_c_^2^)/3
1942 reflections	(Δ/σ)_max_ < 0.001
150 parameters	Δρ_max_ = 0.25 e Å^−^^3^
6 restraints	Δρ_min_ = −0.22 e Å^−^^3^

### N,N'-Bis[tris(hydroxymethyl)methyl]propane-1,3-diamine Fractional atomic coordinates and isotropic or equivalent isotropic displacement parameters (Å^2^)

**Table:**

	*x*	*y*	*z*	*U*_iso_*/*U*_eq_	Occ. (<1)
C1	0.1413 (2)	0.750000	0.6466 (4)	0.0518 (6)	
H1A	0.064 (3)	0.750000	0.554 (5)	0.053 (7)*	
H1B	0.104 (3)	0.750000	0.788 (5)	0.071 (9)*	
C2	0.2155 (2)	0.68773 (9)	0.6187 (3)	0.0546 (5)	
H2A	0.291 (2)	0.6900 (11)	0.706 (4)	0.080 (7)*	
H2B	0.168 (2)	0.6504 (12)	0.675 (4)	0.075 (6)*	
N1	0.25043 (14)	0.67764 (6)	0.4019 (2)	0.0429 (3)	
H1C	0.275 (3)	0.7150 (13)	0.357 (5)	0.043 (9)*	0.5
H1D	0.188 (3)	0.6744 (16)	0.329 (5)	0.033 (8)*	0.5
C3	0.33326 (13)	0.62212 (7)	0.3552 (2)	0.0398 (3)	
C4	0.46486 (15)	0.63221 (9)	0.4434 (3)	0.0510 (4)	
H4A	0.5140 (18)	0.5938 (10)	0.417 (3)	0.050 (5)*	
H4B	0.4579 (17)	0.6395 (9)	0.596 (3)	0.050 (5)*	
O4	0.52710 (14)	0.68472 (8)	0.3514 (3)	0.0724 (5)	
H4C	0.592 (3)	0.6809 (18)	0.284 (6)	0.048 (10)*	0.5
H4D	0.519 (6)	0.7230 (15)	0.375 (9)	0.12 (3)*	0.5
C5	0.28593 (15)	0.55783 (8)	0.4441 (3)	0.0501 (4)	
H5A	0.343 (2)	0.5226 (10)	0.405 (3)	0.059 (5)*	
H5B	0.293 (2)	0.5591 (10)	0.591 (4)	0.062 (6)*	
O5	0.16180 (11)	0.54508 (7)	0.3811 (2)	0.0631 (4)	
H5	0.136 (3)	0.5082 (12)	0.423 (5)	0.103 (9)*	
C6	0.33686 (17)	0.62006 (8)	0.1183 (3)	0.0479 (4)	
H6A	0.2525 (18)	0.6105 (9)	0.072 (3)	0.051 (5)*	
H6B	0.3643 (18)	0.6628 (10)	0.065 (3)	0.058 (5)*	
O6	0.41686 (13)	0.57189 (6)	0.0346 (2)	0.0616 (4)	
H6	0.490 (2)	0.5798 (15)	0.052 (5)	0.104 (10)*	

### N,N'-Bis[tris(hydroxymethyl)methyl]propane-1,3-diamine Atomic displacement parameters (Å^2^)

**Table:**

	*U*^11^	*U*^22^	*U*^33^	*U*^12^	*U*^13^	*U*^23^
C1	0.0506 (12)	0.0636 (15)	0.0410 (12)	0.000	0.0170 (11)	0.000
C2	0.0696 (11)	0.0542 (10)	0.0400 (9)	0.0025 (9)	0.0154 (8)	0.0084 (7)
N1	0.0535 (8)	0.0364 (6)	0.0388 (7)	0.0008 (6)	0.0116 (6)	0.0046 (5)
C3	0.0404 (7)	0.0332 (7)	0.0458 (8)	−0.0037 (6)	0.0032 (6)	0.0031 (6)
C4	0.0406 (8)	0.0500 (9)	0.0625 (11)	−0.0065 (7)	0.0037 (8)	−0.0049 (8)
O4	0.0602 (8)	0.0573 (8)	0.0998 (12)	−0.0234 (7)	0.0339 (8)	−0.0183 (8)
C5	0.0407 (7)	0.0407 (8)	0.0690 (12)	−0.0048 (6)	−0.0056 (8)	0.0159 (8)
O5	0.0443 (6)	0.0509 (7)	0.0941 (11)	−0.0121 (5)	−0.0080 (6)	0.0221 (7)
C6	0.0555 (9)	0.0386 (8)	0.0496 (9)	0.0020 (7)	0.0062 (8)	−0.0051 (7)
O6	0.0526 (7)	0.0546 (7)	0.0776 (9)	0.0017 (6)	0.0052 (7)	−0.0260 (7)

### N,N'-Bis[tris(hydroxymethyl)methyl]propane-1,3-diamine Geometric parameters (Å, º)

**Table:**

C1—C2^i^	1.515 (2)	C4—O4	1.400 (2)
C1—C2	1.515 (2)	C4—H4A	0.96 (2)
C1—H1A	1.02 (3)	C4—H4B	1.00 (2)
C1—H1B	1.00 (3)	O4—H4C	0.82 (3)
C2—N1	1.465 (2)	O4—H4D	0.80 (3)
C2—H2A	0.99 (3)	C5—O5	1.417 (2)
C2—H2B	0.99 (3)	C5—H5A	0.98 (2)
N1—C3	1.4759 (19)	C5—H5B	0.95 (2)
N1—H1C	0.86 (2)	O5—H5	0.85 (2)
N1—H1D	0.82 (2)	C6—O6	1.416 (2)
C3—C5	1.526 (2)	C6—H6A	0.973 (19)
C3—C6	1.532 (2)	C6—H6B	0.99 (2)
C3—C4	1.536 (2)	O6—H6	0.81 (2)
			
C2^i^—C1—C2	114.9 (2)	O4—C4—C3	112.62 (16)
C2^i^—C1—H1A	111.0 (7)	O4—C4—H4A	107.1 (11)
C2—C1—H1A	111.0 (7)	C3—C4—H4A	109.2 (11)
C2^i^—C1—H1B	108.6 (8)	O4—C4—H4B	109.7 (11)
C2—C1—H1B	108.6 (8)	C3—C4—H4B	108.5 (11)
H1A—C1—H1B	102 (2)	H4A—C4—H4B	109.6 (16)
N1—C2—C1	111.53 (16)	C4—O4—H4C	124 (3)
N1—C2—H2A	109.8 (15)	C4—O4—H4D	128 (5)
C1—C2—H2A	108.9 (14)	H4C—O4—H4D	106 (5)
N1—C2—H2B	111.9 (14)	O5—C5—C3	111.35 (14)
C1—C2—H2B	109.7 (13)	O5—C5—H5A	111.9 (12)
H2A—C2—H2B	104.7 (19)	C3—C5—H5A	109.6 (12)
C2—N1—C3	117.32 (14)	O5—C5—H5B	111.2 (14)
C2—N1—H1C	106 (2)	C3—C5—H5B	109.1 (13)
C3—N1—H1C	116 (2)	H5A—C5—H5B	103.4 (18)
C2—N1—H1D	110 (2)	C5—O5—H5	112 (2)
C3—N1—H1D	108 (2)	O6—C6—C3	114.61 (15)
H1C—N1—H1D	98 (3)	O6—C6—H6A	107.8 (11)
N1—C3—C5	112.95 (13)	C3—C6—H6A	106.8 (11)
N1—C3—C6	103.95 (12)	O6—C6—H6B	107.8 (12)
C5—C3—C6	111.15 (14)	C3—C6—H6B	109.4 (12)
N1—C3—C4	111.92 (13)	H6A—C6—H6B	110.4 (16)
C5—C3—C4	106.41 (13)	C6—O6—H6	113 (2)
C6—C3—C4	110.56 (14)		
			
C2^i^—C1—C2—N1	−70.4 (3)	C6—C3—C4—O4	50.48 (19)
C1—C2—N1—C3	174.27 (15)	N1—C3—C5—O5	53.8 (2)
C2—N1—C3—C5	52.1 (2)	C6—C3—C5—O5	−62.6 (2)
C2—N1—C3—C6	172.74 (15)	C4—C3—C5—O5	176.99 (16)
C2—N1—C3—C4	−67.92 (19)	N1—C3—C6—O6	176.97 (13)
N1—C3—C4—O4	−64.88 (19)	C5—C3—C6—O6	−61.23 (19)
C5—C3—C4—O4	171.30 (16)	C4—C3—C6—O6	56.72 (18)

Symmetry code: (i) *x*, −*y*+3/2, *z*.

### N,N'-Bis[tris(hydroxymethyl)methyl]propane-1,3-diamine Hydrogen-bond geometry (Å, º)

**Table:**

*D*—H···*A*	*D*—H	H···*A*	*D*···*A*	*D*—H···*A*
N1—H1*C*···N1^i^	0.86 (2)	2.24 (3)	2.970 (3)	143 (3)
O4—H4*D*···O4^i^	0.80 (3)	1.90 (3)	2.679 (3)	162 (6)
O4—H4*C*···N1^ii^	0.82 (3)	2.09 (3)	2.905 (2)	175 (4)
N1—H1*D*···O4^iii^	0.82 (2)	2.09 (3)	2.905 (2)	169 (3)
O6—H6···O5^ii^	0.81 (2)	2.02 (3)	2.7390 (19)	148 (3)
O5—H5···O6^iv^	0.85 (2)	1.88 (2)	2.7305 (18)	176 (3)

Symmetry codes: (i) *x*, −*y*+3/2, *z*; (ii) *x*+1/2, *y*, −*z*+1/2; (iii) *x*−1/2, *y*, −*z*+1/2; (iv) −*x*+1/2, −*y*+1, *z*+1/2.
